# Contemporary outcomes after pericardial window surgery: impact of operative technique

**DOI:** 10.1186/s13019-016-0466-3

**Published:** 2016-04-26

**Authors:** Sarah E. Langdon, Kristen Seery, Alexander Kulik

**Affiliations:** Lynn Heart and Vascular Institute, Boca Raton Regional Hospital, and Charles E. Schmidt College of Medicine, Florida Atlantic University, Boca Raton, Florida USA

**Keywords:** Pericarditis, Pericardial effusion, Pain, Recurrence

## Abstract

**Background:**

The optimal window procedure for drainage of a large pericardial effusion has yet to be established. The purpose of this study was to compare the outcomes associated with the subxiphoid and thoracotomy pericardial window techniques, with a focus on perioperative pain and effusion recurrence rates.

**Methods:**

A retrospective single-center observational study of all pericardial window operations was performed, with the incision based on surgeon preference. Perioperative data was recorded including time to extubation, narcotic requirements, and the development of a recurrent pericardial effusion.

**Results:**

From 2002 to 2015, 179 patients with a large pericardial effusion underwent either a subxiphoid (*n* = 127) or left anterior mini-thoracotomy (*n* = 52) pericardial window procedure. Patients (mean age 73.2 years, 56 % female) had a high incidence of previous malignancy (49 %), chronic anticoagulation (34 %), recent infection (26 %), or renal failure (18 %). Cardiac tamponade was present in 50 %, and 12 % had undergone previous pericardiocentesis. Comparing the two techniques, there was no difference in the amount of fluid drained or in the perioperative mortality rate. Postoperatively, patients who had the subxiphoid approach required less time before extubation (*P* = 0.002) and needed less narcotics within 48 h after surgery (*P* = 0.0001) compared to thoracotomy patients. However, patients treated with the subxiphoid technique more often developed recurrent moderate or large pericardial effusions (*P* = 0.02), and there was a trend towards more repeat operations needed (*P* = 0.15).

**Conclusion:**

Pericardial window surgery via a subxiphoid incision is associated with less postoperative pain and faster time to extubation. However, the thoracotomy approach may be more effective at preventing effusion recurrence and the need for repeat surgery.

## Background

Several therapies are available for patients with a large pericardial effusion to alleviate symptoms or prevent complications such as cardiac tamponade. Pericardiocentesis is often considered, but this non-operative approach has been abandoned by many since it does not achieve complete fluid evacuation, and it is associated with high recurrence rates [1–3]. With surgery, a pericardial window operation offers definitive management, limiting fluid recurrence by allowing the effusion to continuously drain after a portion of the pericardium is excised [4]. Two common techniques exist for pericardial window surgery, although the optimal procedure has yet to be established [5]. The subxiphoid method involves approaching the pericardium under the xiphoid process of the sternum, whereas the thoracotomy technique comprises access to the pericardium through an incision in the left fifth intercostal space. Conflicting data have been presented regarding the different window options. Some older studies have favored the subxiphoid approach, noting a high rate of respiratory complications with the thoracotomy technique which historically involved lengthy incisions [6]. In the current era however, when smaller incisions are promoted (i.e., mini-thoracotomy), comparative pericardial window data are not available, such as ventilation requirements and pain intensity after surgery. Moreover, it remains unclear whether a “true” pericardial window into the pleural space can improve the long-term durability of window surgery and prevent effusion recurrence [7]. Therefore, the purpose of this study was to evaluate contemporary outcomes after pericardial window surgery and compare the subxiphoid and thoracotomy techniques, with a specific focus on perioperative pain, ventilatory support, and durability, including the need for repeat surgery for effusion recurrence.

## Methods

### Study design

A retrospective single-center observational study of all pericardial window operations was performed. The study cohort was drawn from the electronic medical records of Boca Raton Regional Hospital, leading to the identification of all patients who underwent a pericardial window operation since the inception of the database in 2002. A retrospective chart review was then performed to confirm the use of the surgical procedure. Demographic and clinical information was documented for each patient who underwent a pericardial window operation using either a subxiphoid or left anterior thoracotomy incision from April 2002 to June 2015. Patients were excluded if they were treated with pericardiocentesis only. The current study was approved by the Boca Raton Regional Hospital Research Committee (institutional review board), Boca Raton, Florida.

### Technique

Pericardial window surgery was indicated to treat a moderate or large pericardial effusion in association with symptoms, hypotension, the need for long-term anticoagulation, or for pathologic diagnosis (i.e., suspected metastatic cancer). The incision choice for a pericardial operation was dictated by surgeon preference. Occasionally, factors could favor one technique over another, such as patient body habitus. Both approaches for pericardial window surgery were applied consistently throughout the study time period. The subxiphoid technique involved a 3–5 cm incision over the xiphoid extending into the midline, with division of the linea alba, resection of the xiphoid and upwards/forwards retraction of the sternum. The pericardium was drained and a 2–3 cm pericardial specimen was resected to create the window. A chest tube was then inserted into the pericardial space through a separate stab wound and connected to suction, and the wound was closed in layers.

For the thoracotomy approach, a 3–5 cm incision was created beneath the left nipple, and the chest was entered through a 5th intercostal interspace anterior mini-thoracotomy. The left pleural cavity was drained. The pericardial fluid was then evacuated and a 2–3 cm segment of the pericardium was resected to create the window. Adhesions were divided with sharp and blunt dissection to facilitate careful chest tube placement. In all thoracotomy cases, a chest tube was placed within the pericardial cavity for complete drainage of pericardial space. Usually, a second chest tube was also placed in the left pleural cavity. The chest tubes were passed through separate incisions in the sixth intercostal space and connected to suction prior to closure of the wound.

With either technique, chest tubes were typically kept in position for 48–72 h postoperatively, and they were usually removed when the drainage amount was less than 100 mL over a 24 h period. No sclerosing agents were used. An echocardiogram was not routinely ordered postoperatively early in the series, but in more recent years, nearly all patients underwent postoperative echocardiographic follow-up within 2 weeks, regardless of clinical course (75 % of entire cohort received a postoperative echocardiogram).

### Covariates

Perioperative data was collected through retrospective chart review, with a focus on surgical technique and patient comorbidities. For each patient, recorded preoperative characteristics included: age, gender, infection within 1 month, the use of anticoagulation therapy, history of atrial fibrillation, malignancy, hypertension, diabetes, stroke, end-stage renal disease requiring dialysis, previous pericardiocentesis, recent electrophysiology procedure or perforation (within 1 month), and previous cardiovascular surgery. Echocardiographic data that was collected included pericardial effusion size and the presence cardiac tamponade (right atrial compression or right ventricular diastolic compression or both). Operative data included the surgical technique employed, the amount of fluid drained, and blood pressure response upon fluid evacuation.

### Outcomes

Postoperative data was recorded, including time to extubation, hospital length of stay and the occurrence of postoperative complications including death (within 30 days). The intensity of postoperative pain was assessed by recording all narcotics administered within the first 48 h after surgery, and converting each narcotic (based on type, amount and route) to intravenous morphine equivalents measured in milligrams [8, 9]. This provided a single unit of measure to facilitate pain comparison between groups. Narcotics were administered to control moderate or severe pain early after surgery, and no “standing” or automatic narcotic orders were in place during the time period of the study. Each patient’s medical chart was further reviewed for postoperative echocardiographic data to note the development of a recurrent pericardial effusion and document the need for repeat pericardial window surgery. The size of a postoperative recurrent pericardial effusion was classified as moderate (10–20 mm) or large (>20 mm) based on current echocardiographic guidelines [10]. Clinical follow-up data was available for 100 % of the cohort. The mean follow-up duration for the cohort was 5.4 years (maximum 13 years).

### Statistical analysis

Standard descriptive statistical analyses were used. Continuous data are presented as a mean ± standard deviation and categorical data are presented as proportions. Comparisons between patients who underwent subxiphoid and thoracotomy incisions were performed between the groups using unpaired two-sided Student’s t tests, Chi-squared tests or Fisher’s exact tests. Specifically, the two groups were compared in terms of the amount of fluid drained in the operating room, the time to extubation, the amount of intravenous morphine equivalents required within 48 h, hospital length of stay and perioperative mortality. Moreover, the development of a recurrent moderate or large pericardial effusion and the need for a repeat pericardial window procedure were compared between the two groups. All reported *P* values are two-sided and considered significant if <0.05. All analyses were performed using Stata/MP version 11.2 (StataCorp, College Station, TX).

## Results

### Patient cohort

The study cohort consisted of 179 patients who underwent pericardial window surgery from 2002 to 2015. The mean age of the cohort was 73.2 ± 11.9 years, and 56 % were female. Patients had a high incidence of previous malignancy (49 %), chronic anticoagulation (34 %), recent infection (26 %), or chronic renal failure (18 %). Cardiac tamponade was present in 50 %, and 12 % had undergone previous pericardiocentesis. In the operating room, on average, 493 ± 293 mL of fluid was evacuated from the pericardial cavity. Pericardial fluid analysis from 19 patients noted the presence of malignancy. The average hospital length of stay was 11.0 ± 8.1 days.

Of the total cohort, 127 patients had a subxiphoid operation, and 52 patients had window surgery via the thoracotomy approach. Table 1 describes the characteristics of two groups in the study. Compared to the thoracotomy patients, those treated with the subxiphoid approach more often had a history of previous (cured) malignancy or previous treatment with pericardiocentesis.

**Table 1:** Preoperative Characteristics

Characteristic	Subxiphoid (*N* = 127)	Thoracotomy (*N* = 52)	*P* Value
Age, years	73.6 ± 11.6	72.3 ± 12.8	0.52
Female gender	75 (59.1 %)	26 (50.0 %)	0.32
Body weight, kg	75.4 ± 25.3	77.3 ± 24.6	0.84
Recent infection	36 (28.4 %)	11 (21.2 %)	0.36
Previous (cured) malignancy	49 (38.6 %)	10 (19.2 %)	0.01
Current (ongoing) malignancy	21 (16.6 %)	7 (13.5 %)	0.82
Obstructive lung disease	4 (3.2 %)	1 (1.9 %)	1.00
Previous lung cancer	15 (11.8 %)	5 (9.6 %)	0.80
Anticoagulation	41 (32.3 %)	20 (38.5 %)	0.49
Preoperative atrial fibrillation	46 (36.2 %)	27 (51.9 %)	0.06
Hypertension	79 (62.2 %)	37 (71.2 %)	0.30
Diabetes mellitus	23 (18.1 %)	13 (25.0 %)	0.31
Previous stroke	15 (11.8 %)	6 (11.5 %)	1.00
Renal dysfunction	21 (16.5 %)	11 (21.2 %)	0.52
Recent electrophysiology procedure	19 (15.0 %)	13 (25.0 %)	0.13
Previous cardiac surgery	13 (10.2 %)	7 (13.5 %)	0.60
Previous pericardiocentesis	21 (16.7 %)	1 (2.0 %)	0.01
Echocardiographic features of cardiac tamponade	59 (46.8 %)	30 (58.8 %)	0.18

### Outcome comparison

Table 2 summarizes the clinical outcomes of the patient cohort. In the operating room, patients treated with the two techniques had similar amounts of fluid drained from the pericardial space (intraoperative pericardial fluid drainage: subxiphoid versus thoracotomy, 512 ± 303 mL versus 452 ± 267 mL, *P* = 0.22). Following surgery, patients treated with the subxiphoid approach required less time before extubation (*P* = 0.002). Patients who had a subxiphoid incision also required less narcotics after surgery, suggesting less postoperative pain, compared to the thoracotomy patients (intravenous morphine equivalents within 48 h: subxiphoid versus thoracotomy, 32.4 ± 40.5 mg versus 78.3 ± 73.0 mg, *P* = 0.0001). The perioperative mortality rate (subxiphoid versus thoracotomy, 7.1 % versus 7.7 %, *P* = 1.00) and the hospital length of stay (subxiphoid versus thoracotomy, 11.0 ± 7.5 days versus 11.1 ± 9.5 days, *P* = 0.91) were similar between the two groups. The causes of death after surgery (*N* = 13) appeared unrelated to the incision site, but instead were associated with multiple (and often concurrent) preoperative factors, including severe infection (*N* = 2), metastatic cancer (*N* = 7), and ongoing multi-organ failure (*N* = 9).

**Table 2:** Postoperative Outcomes

Outcome	Subxiphoid (*N* = 127)	Thoracotomy (*N* = 52)	*P* Value
Time to extubation, hours	3.6 ± 10.1	12.9 ± 21.9	0.002
Intravenous morphine equivalents (48 h)	32.4 ± 40.5	78.3 ± 73.0	0.0001
Hospital length of stay, days	11.0 ± 7.5	11.1 ± 9.5	0.91
Postoperative pericardial effusion (moderate or large)	12 (9.4 %)	0 %	0.02
Repeat pericardial window	5 (3.9 %)	0 %	0.15
Perioperative death	9 (7.1 %)	4 (7.7 %)	1.00

Postoperative echocardiograms revealed a significantly greater rate of recurrent moderate or large pericardial effusions with the subxiphoid technique (subxiphoid versus thoracotomy, 9.4 % versus 0 %, *P* = 0.02). Repeat pericardial window surgery was required for 5 symptomatic subxiphoid patients (3.9 %) who developed recurrent pericardial effusions after the initial operation. All repeat operations were performed within 1 month of the initial window surgery. Repeat surgery was not needed for any patient in the thoracotomy group (*P* = 0.15). The presence of malignant cells in the pericardial fluid did not appear to influence the rate of recurrence. Only 1 of the 12 patients who developed a recurrent pericardial effusion had a neoplastic effusion. No patient undergoing repeat surgery had malignant cells in the effusion.

## Discussion

Pericardial disease has been a subject of interest since the times of Hippocrates and Galen [11]. Caused by a multitude of pathological processes, a pericardial effusion can develop secondary to infection, malignancy, and uremia, as well as iatrogenic injury from pacemaker insertion or post-cardiac surgery. Pericardial effusions are rarely symptomatic, and are often incidental findings noted on imaging studies. However, with rapid or extensive fluid accumulation, symptoms and life-threatening hemodynamic consequences may develop. Drainage of the pericardial space to treat cardiac tamponade using a subxiphoid approach was first described in the early 1800’s [11]. Over time however, with the advent of modern thoracic surgery, the subxiphoid technique fell into disuse, and the thoracotomy approach with pericardiectomy or creation of a pericardial window became the treatment of choice. Thereafter, in the early 1970’s, the subxiphoid technique became popular once again for the drainage of effusive pericardial disease [12].

To this day, controversy persists regarding the optimal surgical treatment for a pericardial effusion [13]. The subxiphoid technique has been criticized by some for a higher recurrence rate since it does not involve the creation of a “true” pericardial window into the pleural space [7]. On the other hand, the thoracotomy approach is believed to be a more invasive operation with greater potential for morbidity. While this may relate to the lengthy thoracotomy incisions employed in the past [6], it remains unclear whether the perioperative risk is still higher in the current era with the present use of mini-thoracotomy incisions.

In this study, we sought to compare contemporary outcomes following subxiphoid and thoracotomy window operations, with a focus on perioperative pain, ventilatory support, and durability. At our center, the technique chosen is mainly based on the preference of the operating surgeon. Occasionally however, clinical or anatomic factors may favor one approach over another. For example, should a patient develop acute hypotension on induction of anesthesia, a thoracotomy incision can facilitate faster drainage of the pericardial cavity. The thoracotomy approach may also be useful for a morbidly obese patient, since extensive abdominal adipose tissue can interfere with subxyphoid exposure. Alternatively, the subxyphoid technique may be chosen for a patient who recently underwent sternotomy during a cardiac operation, or if there is a question of whether a full sternotomy may be necessary in an emergency situation to control bleeding (i.e., pacemaker insertion complication).

Our data noted that both techniques were equally effective in terms of intraoperative drainage. However, patients treated with the thoracotomy approach had significantly longer ventilatory support requirements after surgery, and they needed significantly greater amounts of narcotics for pain control in the first 48 h postoperatively. In contrast, subxiphoid patients developed recurrent pericardial effusions significantly more often, and there was a trend towards more repeat window surgery, although the latter did not reach statistical significance. Thus, compared to the subxiphoid technique, the apparent greater durability of the thoracotomy approach came at the price of longer ventilatory support and greater postoperative pain after surgery.

Pericardiocentesis is considered by some to be the first line treatment for symptomatic pericardial effusions that do not respond to anti-inflammatory therapies [2]. A non-operative approach, pericardiocentesis may relieve symptoms and enable some patients to avoid surgery all together. Preoperative pericardiocentesis may also have a role as a method to avoid hemodynamic instability at the time of anesthetic induction immediately before window surgery [7]. Reviewing the literature, it is clear that pericardiocentesis is associated with a greater risk of recurrence compared to a window operation for the management of a large pericardial effusion. Recurrence rates as high as 60 % have been reported with the use of pericardiocentesis in some series [1], although a recent systematic review including 331 patients reported an overall recurrence rate of 13.9 % after percutaneous drainage [5]. Evidently, the management of patients with pericardial effusions varies from center-to-center based on local expertise, experience, and physician preference. However, our institution and others have essentially abandoned the option of pericardiocentesis for patients with pericardial disease because of the high recurrence rates and frequent obstruction of the pericardiocentesis drainage tube due to blood clot and fibrinous debris [1, 2]. Our cardiovascular and oncology teams prefer the durable solution provided by a pericardial window operation and the use of a large caliber drainage tube. Moreover, a pericardial window operation is a relatively low-risk procedure and offers reliable diagnostic capabilities, including tissue biopsy. Importantly, nearly 11 % of patients in this study cohort had evidence of metastatic disease identified in their pericardial fluid or tissue.

The debate regarding the optimal technique for a pericardial window operation has been the focus of several previous investigations in the field. In one of the first comparative studies, Naunheim et al. assessed the outcomes of 78 patients who were treated with transthoracic incisions for a pericardial effusion, as compared to 53 patients who were treated with subxiphoid procedures, between 1979 and 1989. Operative mortality was similar between the groups, but patients treated with the thoracotomy approach had a higher incidence of postoperative respiratory complications such as pneumonia, pleural effusion, prolonged ventilation and the need for reintubation (11 % versus 35 %, subxiphoid versus transthoracic, *P* < 0.005) [6]. Interestingly, reflecting an earlier approach to the management of pericardial effusive disease, 42 of the 78 patients treated with the transthoracic approach received a sternotomy incision, and 50 of the 78 underwent either a partial or complete pericardiectomy. In a more recent study, Liberman et al. compared the outcomes of 78 subxiphoid patients to 113 transthoracic patients who underwent window surgery between 1992 and 2002. The authors found no difference between the two groups in terms of effusion recurrence (3.7 %) or perioperative complications. However, in-hospital mortality was significantly greater for the subxiphoid group (35 % versus 16 %, *P* = 0.003) [4].

To our knowledge, the present study is the first to document the greater need for narcotics and longer ventilatory times after thoracotomy window surgery, compared to the subxiphoid technique. These results are not completely surprising however, as many investigators have previously noted that thoracic incisions lead to larger decrements in pulmonary function that take longer to resolve, as compared to abdominal incisions [6]. Regarding the risk of mortality, several publications on the subject have noted equivalent perioperative and long-term mortality rates associated with the two window techniques, with survival more dependent on pre-existing conditions rather than on the type of the incision employed [6].

Some authors have reported excellent results utilizing the subxiphoid approach for pericardial drainage, citing the safety and less morbid nature of this operation compared to the thoracotomy technique [6]. Nevertheless, despite the longer ventilatory times and greater need for narcotics, we have grown to favor the thoracotomy approach over the years, given the similar perioperative risk noted with either technique, and the higher recurrence rate after subxiphoid operations. In fact, some series have reported recurrence rates as high as 33 % after subxiphoid operations [4, 6]. However, in a summary of published results involving 560 patients, a recurrence rate of only 3.2 % was noted [5], a rate nearly identical to that seen in the current study. The more promising durability associated with the thoracotomy approach may be a reflection of the window created into the pleural space, compared to the subxiphoid approach where the window can be obstructed by bowel, liver or omentum, even when the peritoneum is opened [7]. We believe that, when using the thoracotomy approach, it is important to place a chest tube not only into the pleural space but also directly into the pericardial cavity to facilitate complete fluid evacuation, pericardial space obliteration, and symphysis of the visceral and parietal pericardium.

The results of our study must be interpreted in the context of the limitations inherent in its design. First, the current paper is a retrospective study of relatively modest size that assessed the outcomes of several surgeons. Since the present study was conducted at a single center, our results may not necessarily be generalizable to other cardiac centers with different patient characteristics or alternative approaches for the management of pericardial effusions. For example, some centers have reported success with video-thorascopic pericardial window operations. However, we have preferred the mini-thoracotomy approach since the video-thorascopic technique requires single-lung ventilation and lateral positioning which may lead to hemodynamic instability [7]. Furthermore, postoperative echocardiographic data was available for only 75 % of patients. Therefore the reported recurrence rates may reflect an underestimate, since it is possible that additional patients could have been identified with clinically silent moderate recurrent effusions, had routine postoperative echocardiograms been ordered for all patients. Finally, we did not measure patient pain prospectively (i.e., visual analog scale), but instead postoperative narcotic administration data was collected retrospectively as a surrogate measurement of pain intensity. In general, our conclusions must be tempered by all the biases inherent in a retrospective observational study, and ideally, in the future, a prospective randomized trial comparing different pericardial window techniques could be organized to determine the optimal approach to pericardial disease. Notwithstanding these limitations, we believe our retrospective analysis adds to the small body of literature comparing the thoracotomy and subxiphoid window techniques, and we hope our study stimulates further interest and research in the field.

## Conclusions

In conclusion, pericardial window surgery in the modern era is an effective technique for the drainage of a large pericardial effusion. Compared to the thoracotomy technique, a window performed via a subxiphoid incision leads to less postoperative pain and earlier postoperative extubation. In contrast, the thoracotomy approach may be more effective at preventing effusion recurrence and possibly the need for repeat surgery thereafter.
